# Adolescent cannabinoid exposure induces irritability-like behavior and cocaine cross-sensitization without affecting the escalation of cocaine self-administration in adulthood

**DOI:** 10.1038/s41598-018-31921-5

**Published:** 2018-09-17

**Authors:** Jenni Kononoff, Philippe A. Melas, Marsida Kallupi, Giordano de Guglielmo, Adam Kimbrough, Maria Scherma, Paola Fadda, Denise B. Kandel, Eric R. Kandel, Olivier George

**Affiliations:** 1 Department of Neuroscience, The Scripps Research Institute, La Jolla, CA 92037 USA; 2 Department of Neuroscience, Columbia University, New York, NY 10032 USA; 3 Department of Clinical Neuroscience, Center for Molecular Medicine, Karolinska University Hospital, Karolinska Institute, Stockholm, 17176 Sweden; 4 Department of Biomedical Sciences, Division of Neuroscience and Clinical Pharmacology, University of Cagliari, Cagliari, 09042 Italy; 5 Centre of Excellence “Neurobiology of Dependence”, University of Cagliari, Cagliari, 09042 Italy; 6 Department of Psychiatry, Vagelos College of Physicians and Surgeons and School of Public Health, Columbia University, and New York State Psychiatric Institute, New York, NY 10032 USA; 7 Howard Hughes Medical Institute, Chevy Chase, MD 20815 USA

## Abstract

Cannabis use is typically initiated during adolescence and is a significant risk factor for the development of cocaine use in adulthood. However, no preclinical studies have examined the effects of adolescent cannabinoid exposure on cocaine dependence in adulthood using the escalation model of cocaine self-administration and the assessment of negative emotional states. In the present study, we found that exposure to the cannabinoid receptor agonist WIN55,212-2 (WIN) in adolescence produced irritability-like behavior and psychomotor cross-sensitization to cocaine in adolescence. In adulthood, rats were allowed to self-administer cocaine. The acquisition of cocaine self-administration was lower in rats with adolescent WIN exposure compared with controls. However, both WIN-exposed and control rats escalated their cocaine intake at the same rate, had similar responding under a progressive-ratio schedule of reinforcement, and had similar psychomotor responses to cocaine. Interestingly, the increase in irritability-like behavior that was previously observed in adolescence after WIN exposure persisted into adulthood. Whether the persisting increase in irritability-like behavior after WIN exposure has translational relevance remains to be studied. In summary, these results suggest that psychoactive cannabinoid exposure during adolescence is unlikely to have a major effect on the escalation of cocaine intake or the development of compulsive-like responding *per se* in adulthood in a rat model of cocaine self-administration. However, whether the persisting irritability-like behavior may predispose an individual to mood-related impairments in adulthood or predict such impairments warrants further investigation.

## Introduction

Cannabis is one of the most frequently used psychoactive substances in the world^1^ and is the subject of major debates between proponents of the gateway hypothesis^2^ and advocates of legalization. Proponents of the gateway hypothesis have argued that epidemiological studies indicate that the early use of cannabis is an important risk factor for initiating cocaine use, that cannabis dependence predicts cocaine dependence^3–6^, that cannabis use may be associated with poor cognitive and psychiatric outcomes in adulthood^7–9^, and that major changes in legalization of the possession, sale, and cultivation of cannabis in the United States may exacerbate these poor outcomes by increasing the level of cannabis use in adolescents and young adults^10^. Cannabis use has been increasingly prevalent among adolescents and young adults. Currently, its use exceeds that of tobacco smoking among adolescents in the United States, in which 37.1% of high school seniors in 2017 reported using cannabis within the past year^11^. Advocates of legalization and medicinal use argue that it is unclear whether the relationship between prior cannabis use and later cocaine use or cocaine use disorder is caused by cannabis use *per se* or other drug-associated factors, such as concomitant psychiatric disorders and socioeconomic status^5,12^. However, epidemiological studies cannot establish causal relationships between the pharmacological effects of exposure to cannabis and the development of cocaine use^3,13^.

Preclinical studies provide a controlled way to study causal relationships between early-life cannabinoid exposure and cocaine use, including compulsive-like use, later in life. Previous studies reported that exposure to the cannabinoid receptor agonist WIN55,212-2 (WIN) during adolescence decreased the reactivity of dopaminergic neurons to WIN (i.e., tolerance), produced cross-tolerance to cocaine in adolescence^14^, and produced cross-sensitization to the psychomotor effects of cocaine in adolescence but not in adulthood^15^. This effect appears to be mediated by the modulation of eukaryotic initiation factors in the brain^15^. Such modifications of key neural substrates may reprogram the adolescent brain and make it more susceptible to the later use of other illicit drugs, such as cocaine^2,16^. However, other groups found that prior treatment with either the main psychoactive constituent of cannabis (Δ^9^- tetrahydrocannabinol [THC]) or WIN had no effect on behavioral responses to amphetamine in either adolescence or adulthood^17^. However, in the study by Ellgren *et al*.^17^, cannabinoid exposure lasted only 5 days, the doses of cannabinoid were low, and the animals were injected only once per day. A major limitation of these preclinical studies is the use of an animal model of cocaine exposure (psychomotor sensitization) that reflects neither the direct acquisition of cocaine use (measured by self-administration) nor the compulsive nature of cocaine use disorder (measured by the escalation of self-administration).

To address this issue, we tested the effect of adolescent exposure to the cannabinoid receptor agonist WIN on key addiction-related behaviors using a more complex animal model of drug addiction. The model included measures of irritability-like behavior, which has recently been used as a measure of the negative emotional state in animal models of addiction^18–20^. We also assessed cocaine-induced locomotion (i.e., cross-sensitization) in adolescence and adulthood and the acquisition of cocaine self-administration under conditions of short access (1 h) and long access (6 h; i.e., escalation of cocaine self-administration) in adulthood. The long-access model represents a comprehensive model of human addiction^21^ because it produces the escalation of cocaine intake that is associated with the emergence of negative emotional states^22^ and compulsive-like responding despite adverse consequence^23–26^.

## Materials and Methods

### Animals

Male Sprague-Dawley rats (Charles River, Wilmington, MA, USA; *n* = 18) were housed 2–3 per cage on a reverse 12 h/12 h light/dark cycle (lights off at 8:00 AM) in a temperature (20–22 °C)- and humidity (45–55%)-controlled animal facility with *ad libitum* access to water and food. All of the experiments were designed to minimize animal suffering and the number of animals used. All of the procedures were conducted in adherence to the National Institutes of Health *Guide for the Care and Use of Laboratory Animals* and were approved by the Institutional Animal Care and Use Committee of The Scripps Research Institute. The rats were considered adolescent until postnatal day (PND) 60^27^.

### Drugs

WIN55,212-2 mesylate (Tocris, Minneapolis, MN, USA) was dissolved in vehicle that contained 2% Tween 80, 2% ethanol, and saline and injected intraperitoneally (i.p.) in a volume of 1 ml/kg of body weight. Cocaine HCl (National Institute on Drug Abuse, Bethesda, MD, USA) was dissolved in 0.9% saline (Hospira, Lake Forest, IL, USA) at a dose of 0.5 mg/kg/0.1 ml infusion and self-administered intravenously.

### Drug treatment

**Figure 1:**
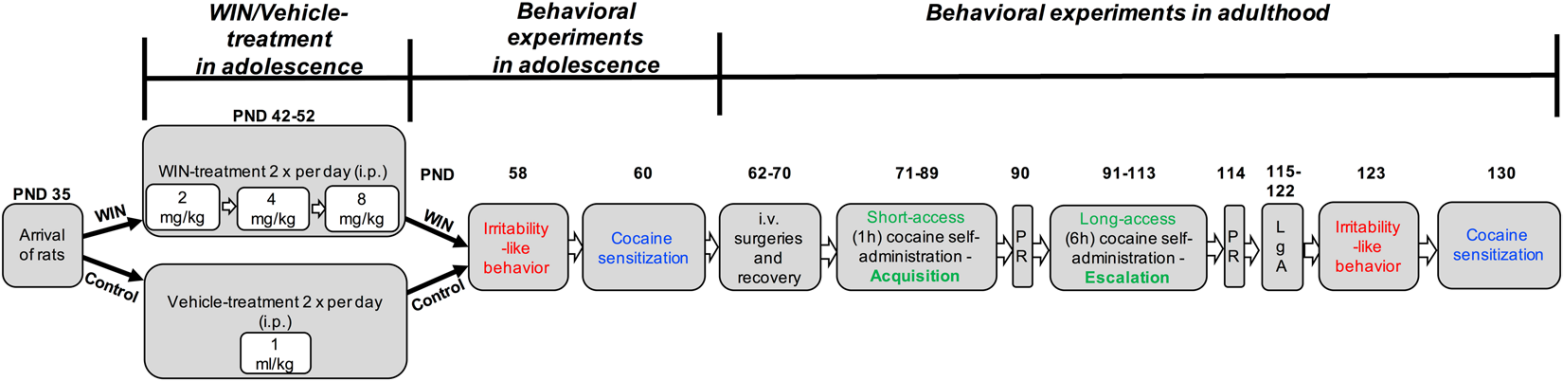
Experimental design of WIN exposure and behavioral testing during adolescence and later in adulthood. LgA, long-access; PND, postnatal day; PR, progressive-ratio schedule of reinforcement; WIN, cannabinoid receptor agonist WIN55,212-2.

The experimental design of WIN exposure and behavioral testing during adolescence and later in adulthood is illustrated in Fig. 1. Prior to treatment, the rats were divided into two groups (*n* = 9/group) that were matched for body weight and food and water intake. These parameters were also monitored throughout WIN treatment. The rats began treatment with WIN or vehicle during adolescence (PND42). Control rats received a corresponding volume of vehicle. Increasing doses of WIN (2 mg/kg, PND42-44; 4 mg/kg, PND45-48; 8 mg/kg, PND49-52) or vehicle were administered twice daily for 11 consecutive days. The WIN dose regimen was chosen according to the literature for subchronic adolescent cannabinoid treatment^28–30^ and because it has been shown to induce psychomotor cross-sensitization to the effects of cocaine in adolescence^15^.

### Irritability-like behavior

All behavioral testing was conducted during the dark phase. To test irritability-like behavior after WIN exposure, we used the bottle-brush test, based on the experimental method that was designed previously for mice^31,32^ and slightly modified to better monitor rat behavior^19^. Currently, this model is increasingly used by both our laboratory and others as a measure of negative emotional states in animal models of addiction^18–20^. This method has advantages over other behavioral paradigms that measure aggressive/defensive behaviors, such as the social dominance/subordination paradigms and resident/intruder confrontation paradigm, because the experimenter has greater control over the mechanical stimulus and thus better precision in ensuring uniform provocation. Furthermore, the “social” factor in eliciting agonistic behavior and the risk of physical injury during an agonistic encounter are both circumvented in the bottle-brush test. The mechanical stimulus of the moving bottle-brush has also been found to be more effective in provoking these behaviors compared with either deceased or stuffed animals^32^.

In the present study, the animals were randomized, and three trained observers scored the rats’ behaviors in real-time as described below. The observers were blinded to treatment of the animals. Testing consisted of ten 10-s trials with 10-s intertrial intervals in plastic cages (27 cm × 48 cm × 20 cm) with clean bedding. A bottle-brush was rotated rapidly toward the rat’s whiskers. Both aggressive responses (smelling, biting, boxing, following, and exploring the bottle-brush) and defensive responses (escaping, digging, jumping, climbing, defecation, vocalization, and grooming) were recorded. The behavioral responses were chosen based on Riittinen *et al*. (1986)^32^ and Lagerspetz and Portin (1968)^33^. Total aggressive and defensive scores were calculated for each animal based on the average score of the observers. Both aggressive and defensive behaviors were summed to calculate the total irritability score. Irritability-like behavior reflects a composite measure of aggressive *vs*. defensive responses. Irritability-like behavior was assessed 6 days after the last injection of WIN/vehicle in adolescence (PND58) and again in adulthood 18 h into withdrawal after the escalation of cocaine self-administration (PND123).

### Cocaine sensitization

Locomotor stimulation by acute cocaine administration was assessed in rats with prior exposure to either vehicle or WIN. This test was performed in adolescence (PND60) to model the previous finding of WIN-induced cocaine cross-sensitization in adolescence^15^ and again in adulthood (PND130). After 30 min habituation to the acrylic experimental chamber (43.2 cm × 43.2 cm × 30.5 cm; Med Associates, St. Albans, VT, USA), the rats were injected with cocaine (10 mg/kg, i.p.), and locomotor activity was recorded for 40 min under red light. Locomotor activity was recorded as the distance traveled (in centimeters) using a video camera that was connected to the ANY-maze Video Tracking System 5.11 (Wood Dale, IL, USA).

### Intravenous catheterization

The rats were anesthetized by isoflurane inhalation, and intravenous catheters were aseptically inserted in the right jugular vein using a modified version of a procedure that was described previously^33–35^. The right jugular vein was punctured with a 22-gauge needle, and the tubing was inserted and secured inside the vein by tying the vein with suture thread. The catheter assembly consisted of an 18 cm length of MicroRenathane tubing (0.023 inch inner diameter, 0.037 inch outer diameter; Braintree Scientific, Braintree, MA, USA) that was attached to a guide cannula (Plastics One, Roanoke, VA, USA). The guide cannula was bent at a near right angle, embedded in dental acrylic, and anchored with a mesh (1 mm thick, 2 cm square). The catheter exited through a small incision on the back, and the base was sealed with a small plastic cap and metal cover cap. The catheters were flushed daily with heparinized saline (10 U/ml of heparin sodium; American Pharmaceutical Partners, Schaumburg, IL, USA) in 0.9% bacteriostatic sodium chloride (Hospira, Lake Forest, IL, USA) that contained 20 mg/0.2 ml of the antibiotic Cefazolin (Hospira, Lake Forest, IL, USA).

### Operant training and escalation of cocaine self-administration in adulthood

Self-administration in adulthood was performed in operant conditioning chambers (Med Associates, St. Albans, VT, USA) that were enclosed in lit, sound-attenuating, ventilated environmental cubicles. The front door and back wall of the chambers were constructed of transparent plastic, and the other walls were opaque metal. Each chamber was equipped with two retractable levers that were located on the front panel. Cocaine was delivered through plastic catheter tubing that was connected to an infusion pump, which was activated by responses on the right (active) lever. Responses on the left (inactive) lever were recorded but did not have any scheduled consequences. Activation of the pump resulted in the delivery of 0.1 ml of cocaine (0.5 mg/kg/0.1 ml). A computer controlled fluid delivery and behavioral data recording. The rats were first trained to self-administer cocaine under a fixed-ratio 1 (FR1) schedule of reinforcement in daily 1-h sessions. Each active lever press resulted in the delivery of one cocaine dose. A 20-s timeout (TO) period followed each cocaine infusion. During the TO period, responses on the active lever did not have scheduled consequences. This TO period occurred concurrently with illumination of a cue light that was located above the active lever to signal delivery of the positive reinforcement. The rats were trained to self-administer cocaine in 14 sessions (5 days/week) until a stable baseline of reinforcement was achieved (≤10% variation over the last three sessions). The criterion for the acquisition of cocaine self-administration was defined as the intake of at least 2.5 mg/kg cocaine in the 1-h self-administration session, requiring at least five lever presses. This criterion was adapted from previous publications^36,37^. After the 14-session acquisition period, the rats were subjected to fourteen 6-h cocaine self-administration sessions to allow them to escalate their cocaine intake^22^. To study the motivation to seek cocaine, a progressive-ratio (PR) schedule of reinforcement was used, in which the response requirement began at one lever press/infusion and increased exponentially according to the following equation^38^: lever presses/infusion = [5 × e^(infusion number × 0.2)^] − 5. The session duration was limited to 6 h or ended when a rat failed to achieve the response requirement within 1 h. The PR sessions were conducted after the training/acquisition phase and again after a stable level of escalation was achieved. In the course of the experiment, which lasted for more than 3 months, some rats were excluded at different stages of the experiment. Two rats (one in the control group and one in the WIN group) were excluded during the acquisition phase because of the failure of catheter patency. In the course of the escalation phase, three rats (one in the control group and two in the WIN group) were excluded from the study because of the failure of catheter patency at the end of the study, and one rat in the vehicle group died unexpectedly during the day off from cocaine self-administration before the study was completed, thus leaving *n* = 6 rats/group for the final analysis. The exclusion of those data did not affect the results of the statistical analysis.

### Statistical analysis

The effects of WIN on body weight and cocaine self-administration were analyzed using two-way repeated-measures analysis of variance (ANOVA). Food and water intake during WIN exposure, irritability-like behavior, 40-min cumulative scores for the cocaine challenge, and the PR data were analyzed using unpaired two-tailed Student’s *t*-test. The acquisition of cocaine self-administration was analyzed using Mantel-Cox survival analysis. Significant effects in the ANOVA were followed by Bonferroni’s multiple-comparison *post hoc* test. Values of *p* < 0.05 were considered statistically significant. The sample size (*n* = 6–9) was chosen based on a *pre hoc* power analysis (Power = 0.8, *p* < 0.05) to detect effect sizes (Cohen’s *d* = 1.3–1.6) that were similar to previously published effect sizes and demonstrated the effect of exposure to cannabinoid receptor agonists on cocaine sensitization (Cohen’s *d* = 1.6)^15^ and cocaine self-administration (Cohen’s *d* = 1.7)^39^. The sample size in the present study was also similar to previous studies that employed the escalation model of drug self-administration^40–44^. The level of intake in the present study was also comparable to previous studies that employed the escalation model of cocaine self-administration with larger sample sizes^34,35,45^.

## Results

### WIN decreases body weight and food intake but increases water intake in adolescence

**Figure 2:**
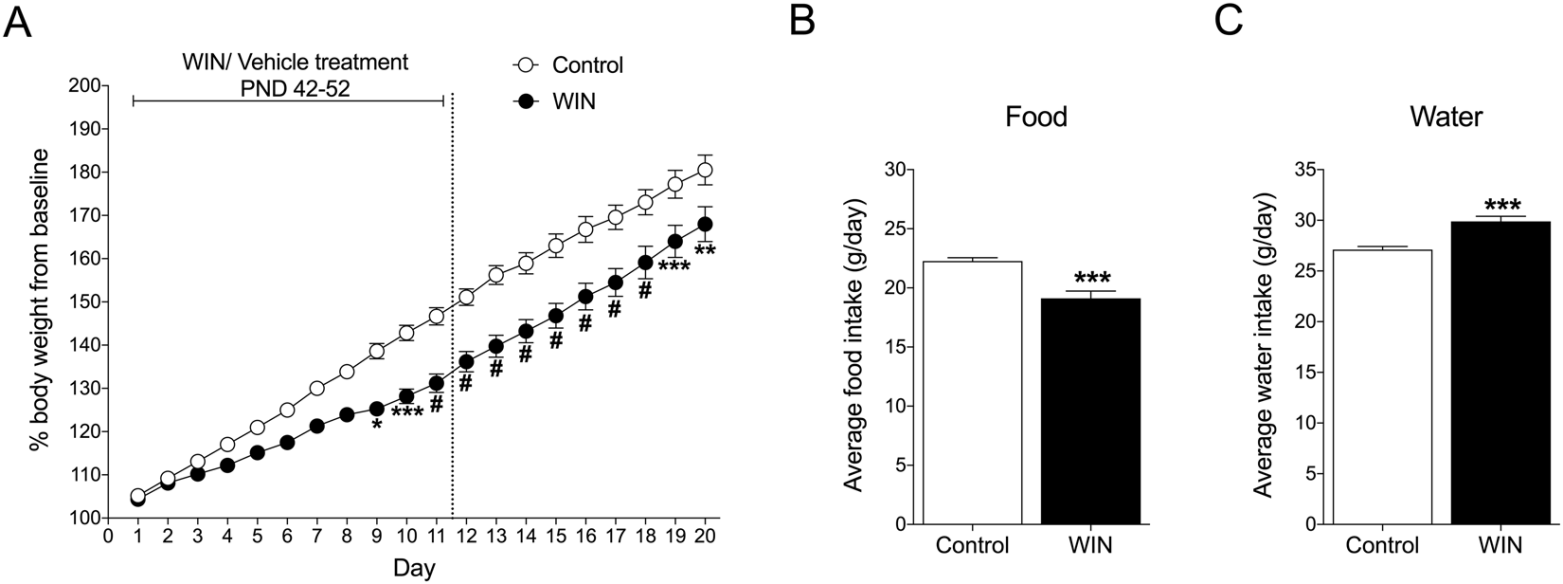
(**A**) Chronic WIN treatment in adolescence decreased body weight. ^*^p < 0.05, ^**^p < 0.01, ^***^p < 0.001; ^#^*p* < 0.0001 (two-way repeated-measures ANOVA followed by Bonferroni’s multiple-comparison *post hoc* test). (**B**) Chronic WIN treatment in adolescence decreased food intake. (**C**) Chronic WIN treatment in adolescence increased water intake. The data are expressed as mean ± SEM. ^***^p < 0.001 (Student’s unpaired two-tailed *t*-test; *n* = 9).

During WIN treatment, body weight and food and water intake were monitored daily. A significant difference in the percentage of body weight gain was observed between WIN- and vehicle-treated rats (Fig. 2A). Weight gain in WIN-treated rats was lower than in vehicle-treated rats, confirmed by a significant interaction (*F*_19,304_ = 8.244, *p* < 0.0001) in the two-way repeated-measures ANOVA. The Bonferroni multiple-comparison *post hoc* analysis revealed that the weight gain difference between groups was significant on days 9–21 (*p* < 0.05 for day 9, *p* < 0.01 for day 20, *p* < 0.001 for days 10 and 19, *p* < 0.0001 for days 11–18). WIN-treated rats also exhibited a reduction of average food intake during WIN treatment compared with controls, confirmed by Student’s two-tailed unpaired *t*-test (*t*_20_ = 4.647, *p* = 0.0002; Fig. 2B). Compared with controls, WIN-treated rats had significantly higher water intake during treatment (*t*_20_ = 4.794, *p* = 0.0001; Fig. 2C).

### WIN induces irritability-like behavior and cross-sensitization to cocaine in adolescence

**Figure 3:**
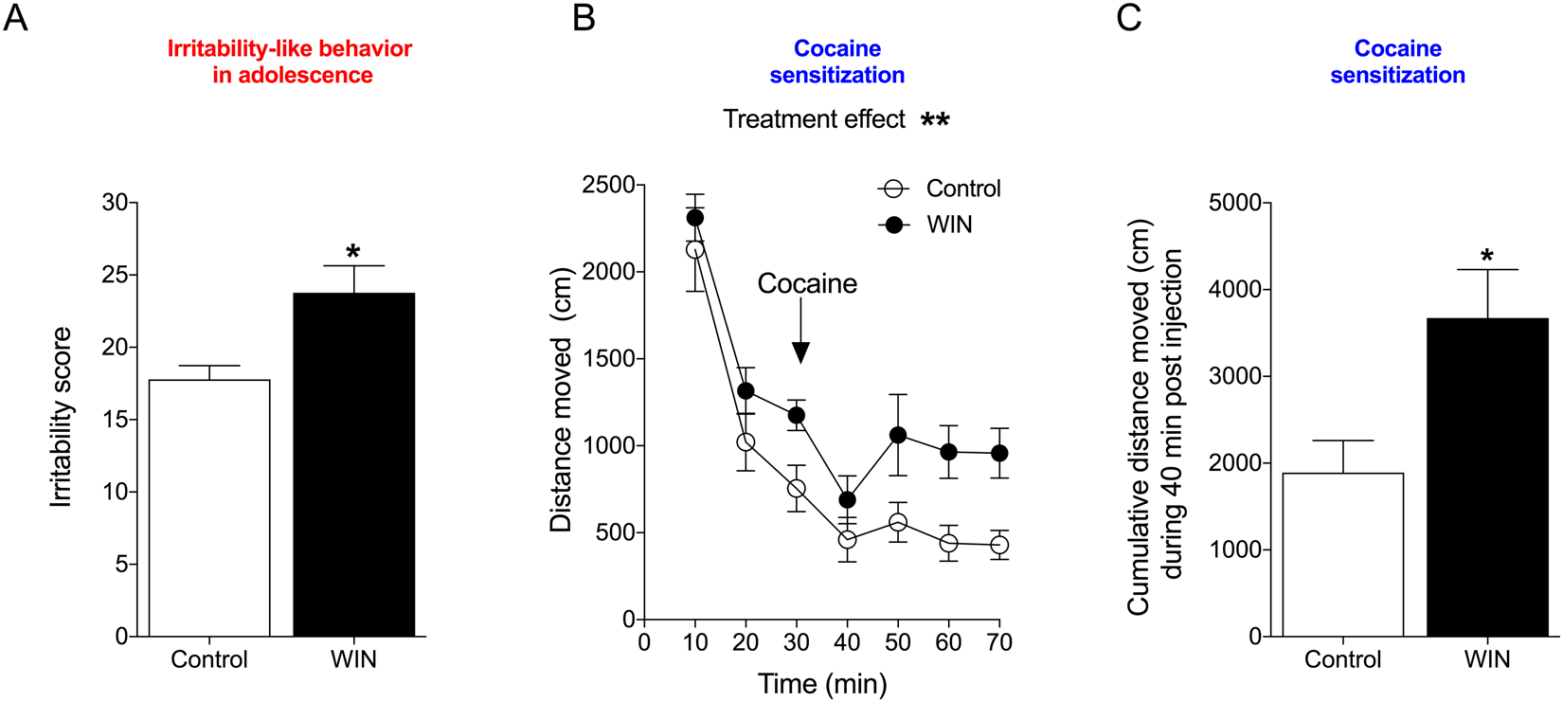
Irritability-like behavior and cocaine-induced cross-sensitization in adolescent rats. (**A**) Irritability-like behavior increases after 6 days of abstinence from WIN. ^*^p < 0.05 (Student’s two-tailed unpaired *t*-test). (**B**) WIN induces behavioral cross-sensitization to the psychostimulant effect of cocaine (10 mg/kg, i.p.). ^**^p < 0.01, significant effect of treatment (two-way repeated-measures ANOVA). (**C**) The 40-min cumulative locomotor activity score during the cocaine challenge demonstrated that WIN-treated rats were more stimulated by cocaine. ^*^p < 0.05 (Student’s two-tailed unpaired *t*-test; *n* = 9).

In adolescence, 6 days after the last WIN/vehicle treatment (PND58), rats that were treated with WIN exhibited an increase in irritability-like behavior compared with controls, measured by the total irritability score. This was analyzed by Student’s two-tailed unpaired *t*-test (*t*_16_ = 2.873, *p* = 0.0111; Fig. 3A). The effect-size and power analyses also confirmed that adolescent WIN treatment increased irritability-like behavior in adolescence (Cohen’s *d* = 1.35, Power = 0.93 at a level of confidence of *p* < 0.05). The individual scores during adolescence for each recorded behavior are listed in Table 1.

**Table 1:** Individual irritability-like behaviors (mean ± SEM) during adolescence and adulthood after WIN exposure.

	ADOLESCENCE				ADULTHOOD			
	Control	WIN	*df* = 16	*p*	Control	WIN	*df* = 10	*p*
Escape	8.4 ± 0.9	8.6 ± 0.6	*t* = 0.2	*p* = 0.8s4	7.4 ± 1.2	8.7 ± 0.4	*t* = 1.0	*p* = 0.34
Digging	0 ± 0	0.7 ± 0.4	*t* = 1.5	*p* = 0.16	0.1 ± 0.1	0.2 ± 0.2	*t* = 0.3	*p* = 0.79
Jumping	0.4 ± 0.3	0.5 ± 0.2	*t* = 0.3	*p* = 0.76	0.2 ± 0.2	0.0 ± 0.0	*t* = 1.0	*p* = 0.34
Climbing	3.4 ± 0.7	4.4 ± 0.5	*t* = 1.3	*p* = 0.21	4.5 ± 1.0	6.3 ± 0.4	*t* = 1.8	*p* = 0.10
Defecation	0.2 ± 0.1	1.3 ± 0.4	*t* = 2.8	*p = 0.01	0.2 ± 0.2	1.2 ± 0.5	*t* = 1.9	*p* = 0.09
Vocalization	0.1 ± 0.1	0.0 ± 0.0	*t* = 1.5	*p* = 0.15	0.1 ± 0.1	0.1 ± 0.1	*t* = 0.5	*p* = 0.66
Grooming	0.3 ± 0.1	0.7 ± 0.4	*t* = 0.8	*p* = 0.42	2 ± 0.9	0.7 ± 0.3	*t* = 1.4	*p* = 0.19
Smelling	1.1 ± 0.2	2.1 ± 0.5	*t* = 1.8	*p* = 0.09	1.4 ± 0.4	2.6 ± 0.7	*t* = 1.6	*p* = 0.14
Biting	0.2 ± 0.1	0.4 ± 0.2	*t* = 0.7	*p* = 0.52	0.0 ± 0.0	0.6 ± 0.5	*t* = 1.34	*p* = 0.24
Boxing	1.8 ± 0.4	1.5 ± 0.4	*t* = 0.5	*p* = 0.60	1.1 ± 0.7	1.7 ± 0.4	*t* = 0.8	*p* = 0.47
Following	1.2 ± 0.4	2.2 ± 0.6	*t* = 1.5	*p* = 0.18	2.4 ± 0.5	3.6 ± 0.7	*t* = 1.3	*p* = 0.21
Exploration	0.7 ± 0.2	1.4 ± 0.4	*t* = 1.7	*p* = 0.10	1.1 ± 0.4	1.7 ± 0.5	*t* = 1.0	*p* = 0.34
Total	17.8 ± 1.0	23.8 ± 1.9	*t* = 2.9	*p = 0.011	20.5 ± 1.9	27.3 ± 2.3	*t* = 2.3	*p = 0.048

On PND60, the rats were placed in the open field for 30 min of habituation, after which they were challenged with cocaine (10 mg/kg, i.p.), and locomotor activity was monitored for 40 min (Fig. 3B). The two-way repeated-measures ANOVA revealed significant effects of treatment (*F*_1,16_ = 10.57, *p* = 0.0050) and time (*F*_6,96_ = 35.71, *p* < 0.0001) but no treatment × time interaction (*F*_6,96_ = 0.5967, *p* = 0.7623). No significant differences were found between groups in cumulative locomotor activity during 30-min habituation in adolescence (*t*_16_ = 1.558, *p* = 0.1387). Rats with prior exposure to WIN exhibited an increase in activity during the cocaine challenge compared with controls, reflected by the 40-min cumulative locomotor activity score during the cocaine challenge (Fig. 3C). This was confirmed by Student’s two-tailed *t*-test (*t*_16_ = 2.655, *p* = 0.0173). The effect-size and power analyses also confirmed that adolescent WIN treatment increased locomotor activity during the cocaine challenge in adolescence (Cohen’s *d* = 1.23, Power = 0.90 at a level of confidence of *p* < 0.05).

### WIN exposure in adolescence decreases the acquisition of cocaine self-administration but has no effect on escalation

**Figure 4:**
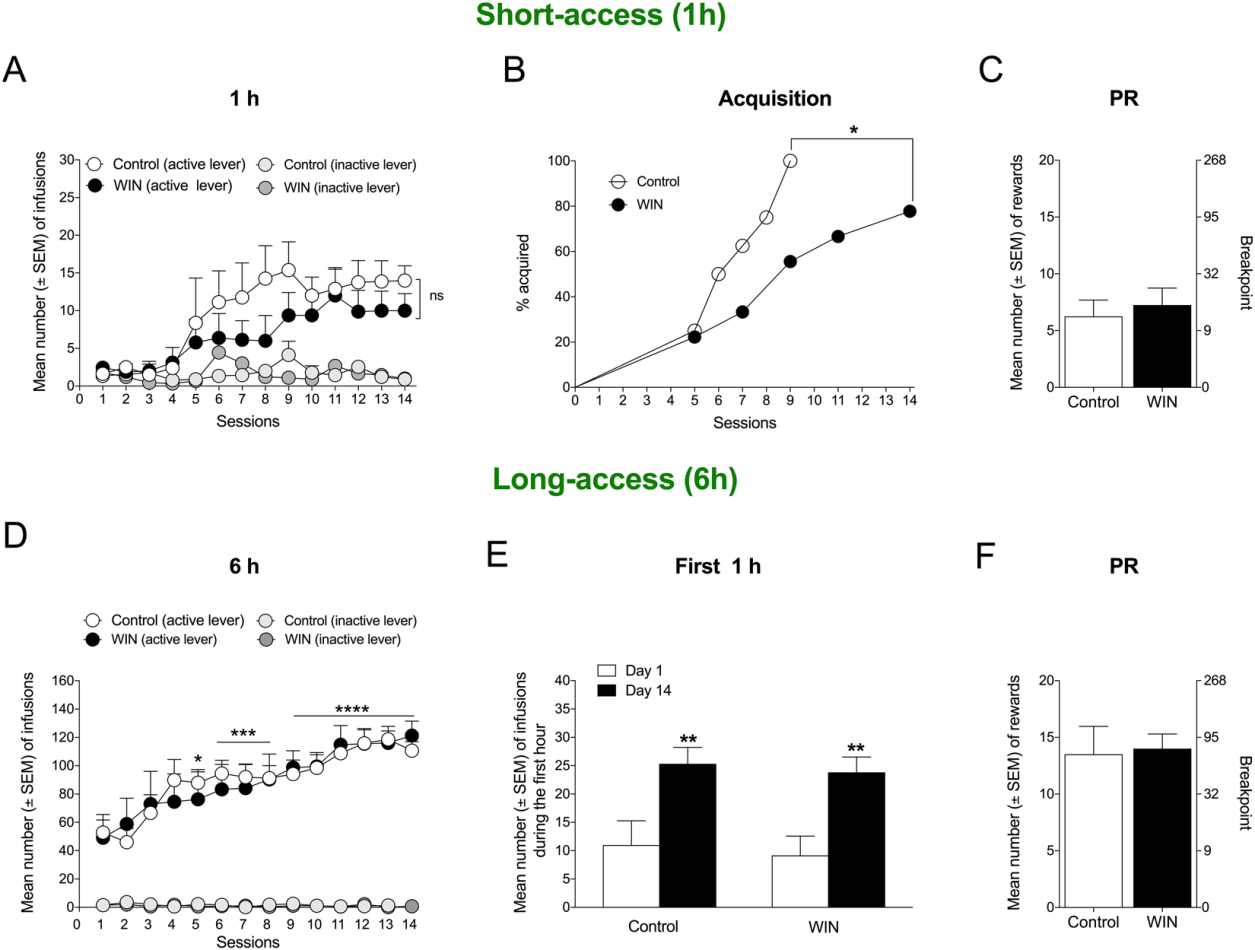
Cocaine self-administration in adulthood. (**A**) Cocaine self-administration on a fixed-ratio 1 (FR1) schedule during daily 1-h sessions (*n* = 8). (**B**) WIN treatment in adolescence decreased the acquisition of cocaine self-administration in adulthood. Acquisition was defined as the first session during which the rat consumed at least 2.5 mg/kg cocaine (five lever presses) in 1 h. ^*^p < 0.05 (Mantel-Cox survival analysis). (**C**) Progressive-ratio (PR) responding for cocaine after short access was unaffected by prior WIN treatment. The data are expressed as the mean ± SEM number of rewards (left y-axis) or breakpoint (right *y*-axis; *n* = 8). (**D**) Prior WIN treatment had no effect on the escalation of cocaine intake during daily 6-h self-administration sessions. ^*^p < 0.05, ^***^p < 0.001, ^****^p < 0.0001, significant difference from baseline (one-way repeated-measures ANOVA followed by Bonferroni’s multiple-comparison *post hoc* test). (**E**) The first hour of the 6-h self-administration session (i.e., the loading phase) demonstrated the significant escalation of cocaine intake during 14 sessions compared with day 1 in both vehicle- and WIN-exposed rats. ^**^p < 0.01 (Student’s two-tailed unpaired *t*-test). (**F**) Progressive-ratio (PR) responding for cocaine after long access (6 h) was unaffected by prior WIN treatment. The data are expressed as the mean ± SEM number of rewards (left y-axis) or breakpoint (right y-axis; *n* = 6).

Nineteen days after the last WIN treatment, the rats had reached adulthood and were trained to self-administer cocaine. Although there was a trend toward lower levels of cocaine self-administration during acquisition (1-h session) in WIN-exposed rats compared with controls, no significant difference was found between controls and WIN-treated rats, confirmed by the lack of an interaction in the two-way repeated-measures ANOVA (*F*_13,182_ = 0.7133, *p* = 0.7488, *n* = 8; Fig. 4A). A significant effect of time was observed (*F*_13,182_ = 0.6285, *p* < 0.0001), demonstrating an increase in self-administration, with no effect of treatment (*F*_1,14_ = 1.208, *p* = 0.2902). However, WIN-treated animals exhibited slower acquisition of cocaine self-administration compared with controls. Only 50% of the WIN-treated rats acquired cocaine self-administration by day 9, whereas 100% of the control rats acquired cocaine self-administration by day 9. The acquisition criterion was a minimum of five lever presses (2.5 mg/kg cocaine) during the short-access session. The significant difference between groups was confirmed by Mantel-Cox survival analysis (log rank *χ*^2^ = 4.211, *p* = 0.0402; Fig. 4B). The motivation to self-administer cocaine at the end of the acquisition period (PND114), measured by PR responding, was unaltered by prior WIN treatment compared with controls (*t*_14_ = 0.482, *p* = 0.6372; Fig. 4C). During 14 long-access (6-h) sessions of cocaine self-administration, both control rats and WIN-treated rats gradually escalated their cocaine intake. The two-way repeated-measures ANOVA confirmed a significant effect of time (*F*_13,130_ = 14.28, *p* < 0.0001) but no effect of treatment (*F*_1,10_ = 0.003261, *p* = 0.9556) and no treatment × time interaction (*F*_13,130_ = 0.5473, *p* = 0.7913). The one-way repeated-measures ANOVA, followed by Bonferroni’s multiple-comparison *post hoc* test, revealed the significant escalation of cocaine self-administration that started in the fifth 6-h session (*p* < 0.05 for session 5, *p* < 0.001 for sessions 6–8, *p* < 0.0001 for sessions 9–14). The two-tailed paired Student’s *t*-test indicated the same level of escalation in the first hour of the 6-h session (i.e., the loading phase) in control rats (*t*_5_ = 6.85, *p* = 0.0010) and WIN-treated rats (*t*_5_ = 5.474, *p* = 0.0028; Fig. 4E). Finally, adolescent WIN treatment had no effect on PR responding after the escalation of cocaine self-administration compared with controls (*t*_10_ = 0.018, *p* = 0.8608; Fig. 4F). The effect-size analysis showed that adolescent WIN treatment had no effect on cocaine escalation (Cohen’s *d* = 0.12), the loading-dose of cocaine self-administration (Cohen’s *d* = 0.22), or compulsive-like responding for cocaine before escalation (Cohen’s *d* = 0.24) or after escalation (Cohen’s *d* = 0.10), measured by PR responding.

### WIN-induced irritability-like behavior but not cocaine cross-sensitization persists into adulthood

**Figure 5:**
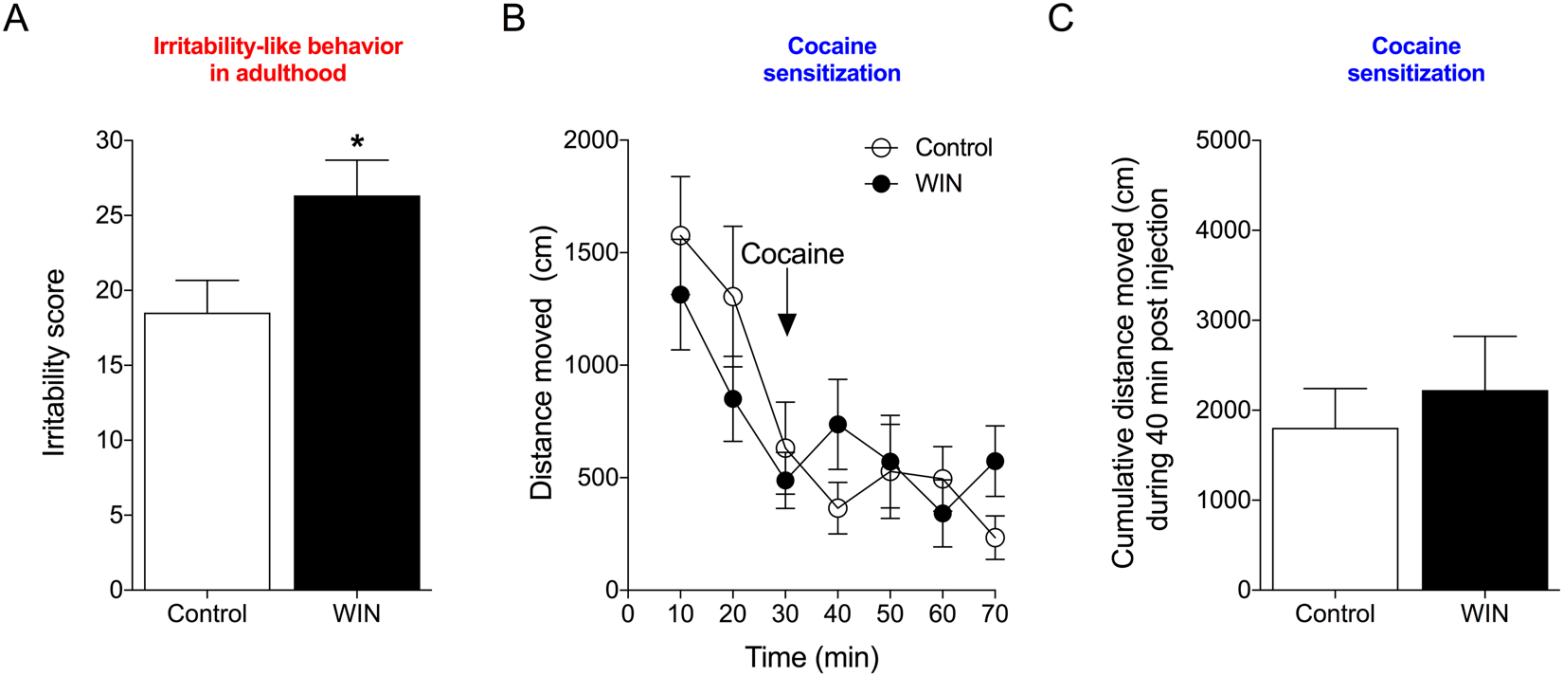
Irritability-like behavior and cocaine-induced locomotor activity in adult rats. (**A**) Prior treatment with WIN increased irritability-like behavior 18 h after the last cocaine self-administration session. ^*^p < 0.05 (Student’s two-tailed unpaired *t*-test). (**B**) Prior WIN treatment did not alter cocaine-induced (10 mg/kg, i.p.) locomotor activity in adulthood. (**C**) The 40-min cumulative locomotor activity score during the cocaine challenge was unaffected by prior WIN treatment. *n* = 6.

Irritability-like behavior was measured again in adulthood after the escalation of cocaine self-administration, 18 h into abstinence from cocaine. The calculated interobserver agreement demonstrated that the observers of behavior were in high agreement (*r* = 0.9350). In the control group, the baseline irritability score before cocaine self-administration was 17.8 ± 0.96 (mean ± SEM). After the escalation of cocaine self-administration, the irritability score was 20.5 ± 1.9, confirming that cocaine withdrawal *per se* did not affect irritability-like behavior. However, in rats with adolescent exposure to WIN, the increase in irritability-like behavior persisted into adulthood (27.3 ± 2.3). The increase in the total irritability score was confirmed by Student’s two-tailed unpaired *t*-test (*t*_10_ = 2.253, *p* = 0.048; Fig. 5A). The effect-size and power analyses also confirmed that adolescent WIN treatment increased irritability-like behavior in adulthood (Cohen’s *d* = 1.42, Power = 0.89 at a level of confidence of *p* < 0.05). The individual scores for each recorded behavior during adulthood are listed in Table 1.

Six days after the last cocaine self-administration session, the rats were again placed in the open field for 30 min of habituation, after which they were challenged with cocaine (10 mg/kg, i.p.), and locomotor activity was monitored for 40 min (Fig. 5B). The two-way repeated-measures ANOVA revealed a significant effect of time (*F*_6,60_ = 11.93, *p* < 0.0001) but no effect of treatment (*F*_1,10_ = 0.04206, *p* = 0.8416) and no treatment × time interaction (*F*_6,60_ = 1.82, *p* = 0.1103). No significant differences were found between groups in cumulative locomotor activity during 30-min habituation in adulthood (*t*_10_ = 0.9881, *p* = 0.3464). Rats with prior exposure to WIN exhibited no difference in 40-min cumulative locomotor activity scores during the cocaine challenge compared with control rats (*t*_10_ = 0.5659, *p* = 0.5839; Fig. 5C). The effect-size analysis also confirmed that adolescent WIN treatment had no effect on locomotor activity during the cocaine challenge in adulthood (Cohen’s *d* = 0.33).

At the end of the escalation phase, the mean ± SEM body weight was 432.5 ± 22.9 g in the control group and 400.83 ± 29.4 g in the WIN group, demonstrating that the WIN-induced reduction of body weight during adolescence recovered in adulthood.

## Discussion

The present study found that adolescent WIN exposure (*i*) increased irritability-like behavior in adolescence, which persisted into adulthood, (*ii*) induced cross-sensitization to the locomotor-stimulating effect of cocaine in adolescence, which did not persist into adulthood, (*iii*) decreased the speed of acquisition but not the rate of cocaine self-administration in adulthood, and (*iv*) had no effect on the escalation of cocaine self-administration in adulthood. Overall, these results demonstrate that although cannabinoid exposure in adolescence induces irritability-like behavior and cross-sensitization to the psychostimulant effect of cocaine during adolescence, it does not promote cocaine self-administration once the animals reach adulthood. However, the effect of adolescent WIN exposure on cocaine self-administration in adolescence was not investigated in the present study because the animals reached adulthood by the time they had recovered from the surgeries that were required for self-administration.

Reductions of both body weight and food intake were observed during WIN treatment. Although the activation of cannabinoid receptors typically produces an increase in food intake in adulthood^46^, accumulating evidence suggests that adolescent exposure to THC or WIN in rats decreases food intake and body weight^15,28,29,47^. The increase in water intake during WIN exposure in the present study confirms the role of cannabinoid receptors in homeostatic responses that regulate not only energy homeostasis but also fluid balance^48^.

Irritability, anxiety, and dysphoria are key negative emotional states that characterize the withdrawal syndrome in humans, which arises when access to the drug is prevented and contributes to drug relapse^49^. Irritability has also been reported to be greater in adolescents at higher risk for substance use^50^. Irritability-like behavior has also been shown to increase during withdrawal from alcohol and nicotine in rodents^18–20^. However, to our knowledge, whether early exposure to cannabinoids affects irritability-like behavior has not been studied in animal models. In the present study, we found that WIN exposure induced irritability-like behavior in adolescence and adulthood, suggesting that cannabinoid exposure in adolescence induces long-lasting neurobehavioral adaptations that can persist months after WIN exposure. However, further studies are needed to investigate whether this finding has translational relevance. An alternative explanation is that, despite blind randomization of the subjects to the two groups, the increase in irritability-like behavior that was observed in WIN-treated rats may be attributable to preexisting differences in irritability-like behavior. Further studies are needed to investigate whether this finding has translational relevance. Numerous human studies demonstrate that early cannabis use is associated with greater vulnerability to the later development of drug addiction and psychiatric illness^51^. A recent study reported a pivotal role for cannabinoid receptors as molecular mediators of adolescent behavior and suggested that cannabinoid receptors may be important in adolescent-onset mental health disorders^52^. Chronic adolescent exposure to WIN has also been shown to induce anxiety-like behavior in rats^53^. However, contradictory findings have also been published, with either no change^54^ or even a decrease^55^ in anxiety-like behavior after cannabinoid exposure in adolescence. Rats that were exposed to cannabis smoke were also reported to exhibit a decrease in anxiety-like behavior^56^. Interestingly, a previous study also demonstrated that long-term cognitive and behavioral dysfunction that was induced by adolescent THC exposure could be prevented by concurrent cannabidiol treatment^57^. Importantly, WIN acts as a full cannabinoid receptor agonist, in contrast to THC, which only acts as a partial agonist. Moreover, cannabis is known to consist of dozens of additional phytocannabinoids apart from THC. Furthermore, different strains of cannabis differ in their THC content, and THC levels in cannabis have increased year after year because of consumer demand, thus making direct comparisons of human data across time and across studies difficult. Nevertheless, we chose this model of early cannabinoid exposure and followed it precisely because it has been shown to induce cocaine cross-sensitization, thus supporting the gateway hypothesis^15^. Further studies are needed to investigate whether the long-term irritability-like behavior that was observed in the present study can be prevented by concurrent cannabidiol treatment or whether adolescent exposure to cannabis smoke induces long-lasting irritability-like behavior in rats.

Epidemiological data consistently document that cannabis exposure precedes the use of other illicit drugs^6,12^. However, epidemiological data cannot provide causal evidence of this sequence. Animal models are particularly useful for studying effects that are related to cross-sensitization because they allow sequential administrations of the studied drugs while controlling for confounding variables. Several studies have reported behavioral cross-sensitization between cannabinoids and stimulants in rodents^58–61^. WIN treatment during adolescence in rats induces long-lasting cross-tolerance to morphine, cocaine, and amphetamine^14^, potentiates amphetamine-induced psychomotor sensitization^62^, and induces cocaine-induced psychomotor sensitization in adolescence^15^. WIN exposure also leads to increases in methylenedioxymethamphetamine-induced^63^ and cocaine-induced^64^ conditioned place preference. In the present study, WIN exposure in adolescence induced cross-sensitization to the stimulatory effect of cocaine in adolescence. However, this effect was no longer present in adulthood when the rats had self-administered cocaine for several weeks, suggesting that cannabinoid exposure in adolescence may increase the psychomotor effects of cocaine during the first exposure to cocaine, but this effect is not necessarily long-lasting.

Cannabinoid exposure increased irritability-like behavior and the psychomotor effects of cocaine, but it did not promote the acquisition or escalation of cocaine self-administration. Indeed, we observed the slower acquisition of cocaine self-administration with 1-h short-access to cocaine in male rats with prior exposure to WIN compared with controls. In contrast, a previous study reported a trend toward an increase in cocaine self-administration during the short (7-day) acquisition phase (30-min session) in female rats with prior exposure to the cannabinoid receptor agonist CP55,940 but not in male rats^39^. However, this study did not discriminate between inactive and active levers, and no difference in cocaine self-administration was observed during the 14-day maintenance phase (2-h session) in either sex^39^. A recent study showed that adolescent WIN exposure caused impairments in an attentional set-shifting task, a measure of cognitive flexibility, in adulthood (PND > 85)^62^. An alternative hypothesis is that the slower acquisition of cocaine self-administration in adulthood that was observed in the present study may be attributable to cognitive impairment that slows the acquisition of operant responding. In humans, several studies have indicated that the adolescent use of cannabis can lead to long-term cognitive deficits, including problems with attention and memory^65^. During escalation, no differences were observed between the rats that were exposed to vehicle in adolescence and the rats that were exposed to WIN in adolescence. This suggests that if cognitive impairments affected the initial acquisition of self-administration, then they did not produce long-term deficits.

The model of long-access to cocaine self-administration is one of the most validated animal models of cocaine use disorder and drug addiction in general^21,22,66^. This model has been shown to result in all seven of the diagnostic criteria of the *Diagnostic and Statistical Manual of Mental Disorders*, 4th edition (DSM-IV), and seven of the 11 DSM-5 criteria, including most of the criteria that are required for severe use disorder: (*i*) tolerance^67^, (*ii*) withdrawal^68,69^, (*iii*) substance taken in larger amount than intended^22^, (*iv*) unsuccessful efforts to quit^70,71^, (*v*) considerable time spent to obtain the drug^72^, (*vi*) important social, work, or recreational activities given up because of use^26,69,73^, and (*vii*) continued use despite adverse consequences^23–25,69,71,74,75^. The present study found no effect of adolescent cannabinoid exposure in the escalation model, suggesting that adolescent WIN exposure may not facilitate the acquisition, maintenance, or escalation of cocaine use in adulthood. An alternative hypothesis is that the effect of cannabinoid use may not be observed on cocaine intake *per se*; instead, cannabinoid exposure may produce an increase in the motivation for cocaine, leading to an increase in compulsive cocaine seeking. Indeed, prior exposure to another potential gateway drug, alcohol, was found to have no effect on subsequent cocaine self-administration *per se* but produced greater motivation and compulsive-like cocaine seeking under a PR schedule of reinforcement^76^. However, we observed no differences between the WIN-exposed and control groups in adulthood when we used a PR schedule of reinforcement to examine whether rats with prior exposure to WIN express alterations of the motivation to self-administer cocaine.

One limitation of long-term behavioral studies in adolescent rats, including the present study, is that puberty in rats is relatively short (~PND38-60 in males)^27^. Compared with adults, rats that are allowed to self-administer cocaine during adolescence (PND42-54) have been shown to be more vulnerable to cocaine addiction^77^. Unfortunately, in the model of cannabinoid exposure during adolescence (PND42-52), cocaine self-administration can only be studied starting in late adolescence and continuing into adulthood because rats exit puberty by PND60^27^. Because of this limitation, one possibility is that cannabinoid exposure during adolescence may affect cocaine intake in adolescence.

The present results demonstrate that chronic exposure to cannabinoids does not facilitate the acquisition of cocaine self-administration or compulsive-like cocaine intake in adulthood, measured by the escalation of cocaine self-administration and PR responding in a relevant model of cocaine use disorder. These results suggest that cannabinoid exposure *per se* is unlikely to be causally responsible for the association between prior cannabis use and future cocaine use in adulthood as purported by the gateway hypothesis. However, we found that cannabinoid exposure produced long-lasting increases in irritability-like behavior, which may indirectly facilitate the emergence of social conflicts and other mental disorders that may contribute to the abuse of drugs other than cocaine. Additionally, the cross-sensitization between WIN and cocaine in adolescence—which was not observed in adulthood—may highlight a short-term increase in the vulnerability to cocaine-induced behaviors.

In summary, the present results showed that cannabinoid exposure during adolescence in rats produced cross-sensitization to cocaine in adolescence and a long-lasting increase in irritability-like behavior in adulthood. However, it did not facilitate the acquisition or escalation of cocaine self-administration or compulsive-like responding for cocaine in adulthood.

## Data Availability

The datasets that were generated and analyzed in the present study are available from the corresponding author upon reasonable request.
